# Why does peer instruction benefit student learning?

**DOI:** 10.1186/s41235-020-00218-5

**Published:** 2020-04-09

**Authors:** Jonathan G. Tullis, Robert L. Goldstone

**Affiliations:** 1 Department of Educational Psychology, University of Arizona, 1430 E. Second St., Tucson, AZ 85721 USA; 2 Department of Psychology, Indiana University, Bloomington, IN USA

**Keywords:** Group decisions, Peer instruction, Metacognition, Confidence, Decision making

## Abstract

In peer instruction, instructors pose a challenging question to students, students answer the question individually, students work with a partner in the class to discuss their answers, and finally students answer the question again. A large body of evidence shows that peer instruction benefits student learning. To determine the mechanism for these benefits, we collected semester-long data from six classes, involving a total of 208 undergraduate students being asked a total of 86 different questions related to their course content. For each question, students chose their answer individually, reported their confidence, discussed their answers with their partner, and then indicated their possibly revised answer and confidence again. Overall, students were more accurate and confident after discussion than before. Initially correct students were more likely to keep their answers than initially incorrect students, and this tendency was partially but not completely attributable to differences in confidence. We discuss the benefits of peer instruction in terms of differences in the coherence of explanations, social learning, and the contextual factors that influence confidence and accuracy.

## Significance

Peer instruction is widely used in physics instruction across many universities. Here, we examine how peer instruction, or discussing one’s answer with a peer, affects students’ decisions about a class assignment. Across six different university classes, students answered a question, discussed their answer with a peer, and finally answered the question again. Students’ accuracy consistently improved through discussion with a peer. Our peer instruction data show that students were hesitant to switch away from their initial answer and that students did consider both their own confidence and their partner’s confidence when making their final decision, in accord with basic research about confidence in decision making. More broadly, the data reveal that peer discussion helped students select the correct answer by prompting them to create new knowledge. The benefit to student accuracy that arises when students discuss their answers with a partner is a “process gain”, in which working in a group yields better performance than can be predicted from individuals’ performance alone.

Peer instruction is specific evidence-based instructional strategy that is well-known and widely used, particularly in physics (Henderson & Dancy, 2009). In fact, peer instruction has been advocated as a part of best methods in science classrooms (Beatty, Gerace, Leonard, & Dufresne, 2006; Caldwell, 2007; Crouch & Mazur, 2001; Newbury & Heiner, 2012; Wieman et al., 2009) and over a quarter of university physics professors report using peer instruction (Henderson & Dancy, 2009). In peer instruction, instructors pose a challenging question to students, students answer the question individually, students discuss their answers with a peer in the class, and finally students answer the question again. There are variations of peer instruction in which instructors show the class’s distribution of answers before discussion (Nielsen, Hansen-Nygård, & Stav, 2012; Perez et al., 2010), in which students’ answers are graded for participation or for correctness (James, 2006), and in which instructors’ norms affect whether peer instruction offers opportunities for answer-seeking or for sense-making (Turpen & Finkelstein, 2007).

Despite wide variations in its implementation, peer instruction consistently benefits student learning. Switching classroom structure from didactic lectures to one centered around peer instruction improves learners’ conceptual understanding (Duncan, 2005; Mazur, 1997), reduces student attrition in difficult courses (Lasry, Mazur, & Watkins, 2008), decreases failure rates (Porter, Bailey-Lee, & Simon, 2013), improves student attendance (Deslauriers, Schelew, & Wieman, 2011), and bolsters student engagement (Lucas, 2009) and attitudes to their course (Beekes, 2006). Benefits of peer instruction have been found across many fields, including physics (Mazur, 1997; Pollock, Chasteen, Dubson, & Perkins, 2010), biology (Knight, Wise, & Southard, 2013; Smith, Wood, Krauter, & Knight, 2011), chemistry (Brooks & Koretsky, 2011), physiology (Cortright, Collins, & DiCarlo, 2005; Rao & DiCarlo, 2000), calculus (Lucas, 2009; Miller, Santana-Vega, & Terrell, 2007), computer science (Porter et al., 2013), entomology (Jones, Antonenko, & Greenwood, 2012), and even philosophy (Butchart, Handfield, & Restall, 2009). Additionally, benefits of peer instruction have been found at prestigious private universities, two-year community colleges (Lasry et al., 2008), and even high schools (Cummings & Roberts, 2008). Peer instruction benefits not just the specific questions posed during discussion, but also improves accuracy on later similar problems (e.g., Smith et al., 2009).

One of the consistent empirical hallmarks of peer instruction is that students’ answers are more frequently correct following discussion than preceding it. For example, in introductory computer science courses, post-discussion performance was higher on 70 out of 71 questions throughout the semester (Simon, Kohanfars, Lee, Tamayo, & Cutts, 2010). Further, gains in performance from discussion are found on many different types of questions, including recall, application, and synthesis questions (Rao & DiCarlo, 2000). Performance improvements are found because students are more likely to switch from an incorrect answer to the correct answer than from the correct answer to an incorrect answer. In physics, 59% of incorrect answers switched to correct following discussion, but only 13% of correct answers switched to incorrect (Crouch & Mazur, 2001). Other research on peer instruction shows the same patterns: 41% of incorrect answers are switched to correct ones, while only 18% of correct answers are switched to incorrect (Morgan & Wakefield, 2012). On qualitative problem-solving questions in physiology, 57% of incorrect answers switched to correct after discussion, and only 7% of correct answers to incorrect (Giuliodori, Lujan, & DiCarlo, 2006).

There are two explanations for improvements in pre-discussion to post-discussion accuracy. First, switches from incorrect to correct answers may be driven by selecting the answer from the peer who is more confident. When students discuss answers that disagree, they may choose whichever answer belongs to the more confident peer. Evidence about decision-making and advice-taking substantiates this account. First, confidence is correlated with correctness across many settings and procedures (Finley, Tullis, & Benjamin, 2010). Students who are more confident in their answers are typically more likely to be correct. Second, research examining decision-making and advice-taking indicates that (1) the less confident you are, the more you value others’ opinions (Granovskiy, Gold, Sumpter, & Goldstone, 2015; Harvey & Fischer, 1997; Yaniv, 2004a, 2004b; Yaniv & Choshen-Hillel, 2012) and (2) the more confident the advisor is, the more strongly they influence your decision (Kuhn & Sniezek, 1996; Price & Stone, 2004; Sah, Moore, & MacCoun, 2013; Sniezek & Buckley, 1995; Van Swol & Sniezek, 2005; Yaniv, 2004b). Consequently, if students simply choose their final answer based upon whoever is more confident, accuracy should increase from pre-discussion to post-discussion. This explanation suggests that switches in answers should be driven entirely by a combination of one’s own initial confidence and one’s partner’s confidence. In accord with this confidence view, Koriat (2015) shows that an individual’s confidence typically reflects the group’s most typically given answer. When the answer most often given by group members is incorrect, peer interactions amplify the selection of and confidence in incorrect answers. Correct answers have no special draw. Rather, peer instruction merely amplifies the dominant view through differences in the individual’s confidence.

In a second explanation, working with others may prompt students to verbalize explanations and verbalizations may generate new knowledge. More specifically, as students discuss the questions, they need to create a common representation of the problem and answer. Generating a common representation may compel students to identify gaps in their existing knowledge and construct new knowledge (Schwartz, 1995). Further, peer discussion may promote students’ metacognitive processes of detecting and correcting errors in their mental models. Students create more new knowledge and better diagnostic tests of answers together than alone. Ultimately, then, the new knowledge and improved metacognition may make the correct answer appear more compelling or coherent than incorrect options. Peer discussion would draw attention to coherent or compelling answers, more so than students’ initial confidence alone and the coherence of the correct answer would prompt students to switch away from incorrect answers. Similarly, Trouche, Sander, and Mercier (2014) argue that interactions in a group prompt argumentation and discussion of reasoning. Good arguments and reasoning should be more compelling to change individuals’ answers than confidence alone. Indeed, in a reasoning task known to benefit from careful deliberation, good arguments and the correctness of the answers change partners’ minds more than confidence in one’s answer (Trouche et al., 2014). This explanation predicts several distinct patterns of data. First, as seen in prior research, more students should switch from incorrect answers to correct than vice versa. Second, the intrinsic coherence of the correct answer should attract students, so the likelihood of switching answers would be predicted by the correctness of an answer above and beyond differences in initial confidence. Third, initial confidence in an answer should not be as tightly related to initial accuracy as final confidence is to final accuracy because peer discussion should provide a strong test of the coherence of students’ answers. Fourth, because the coherence of an answer is revealed through peer discussion, student confidence should increase more from pre-discussion to post-discussion when they agree on the correct answers compared to agreeing on incorrect answers.

Here, we examined the predictions of these two explanations of peer instruction across six different classes. We specifically examined whether changes in answers are driven exclusively through the confidence of the peers during discussion or whether the coherence of an answer is better constructed and revealed through peer instruction than on one’s own. We are interested in analyzing cognitive processes at work in a specific, but common, implementation of classroom-based peer instruction; we do not intend to make general claims about all kinds of peer instruction or to evaluate the long-term effectiveness of peer instruction. This research is the first to analyze how confidence in one’s answer relates to answer-switching during peer instruction and tests the impact of peer instruction in new domains (i.e., psychology and educational psychology classes).

## Method

### Participants

Students in six different classes participated as part of their normal class procedures. More details about these classes are presented in Table 1. The authors served as instructors for these classes. Across the six classes, 208 students contributed a total of 1657 full responses to 86 different questions.

**Table 1:** Descriptions of classes used

Class	Year	Level	Number of Students	Number of Questions	Location
Cognitive Psych (Psych)	2015	Middle level undergrad	61	4	Indiana University
Cognitive Psych (Psych)	2017	Middle level undergrad	60	4	Indiana University
Decision Making (Ed Psych)	2016	Upper level undergrad	24	15	University of Arizona
Decision Making (Ed Psych)	2017	Upper level undergrad	37	16	University of Arizona
Learning Theories (Ed Psych)	2016	Intro Master’s level	12	26	University of Arizona
Learning Theories (Ed Psych)	2018	Intro Master’s level	14	21	University of Arizona

### Materials

The instructors of the courses developed multiple-choice questions related to the ongoing course content. Questions were aimed at testing students’ conceptual understanding, rather than factual knowledge. Consequently, questions often tested whether students could apply ideas to new settings or contexts. An example of a cognitive psychology question used is: Which is a fixed action pattern (not a reflex)?

- Knee jerks up when patella is hit
- Male bowerbirds building elaborate nests [correct]
- Eye blinks when air is blown on it
- Can play well learned song on guitar even when in conversation

### Procedure

The procedures for peer instruction across the six different classes followed similar patterns. Students were presented with a multiple-choice question. First, students read the question on their own, chose their answer, and reported their confidence in their answer on a scale of 1 “Not at all confident” to 10 “Highly confident”. Students then paired up with a neighbor in their class and discussed the question with their peer. After discussion, students answered the question and reported the confidence for a second time. The course instructor indicated the correct answer and discussed the reasoning for the answer after all final answers had been submitted. Instruction was paced based upon how quickly students read and answered questions. Most student responses counted towards their participation grade, regardless of the correctness of their answer (the last question in each of the cognitive psychology classes was graded for correctness).

There were small differences in procedures between classes. Students in the cognitive psychology classes input their responses using classroom clickers, but those in other classes wrote their responses on paper. Further, students in the cognitive psychology classes explicitly reported their partner’s answer and confidence, while students in other classes only reported the name of their partner (the partners’ data were aligned during data recording). The cognitive psychology students then were required to mention their own answer and their confidence to their partner during peer instruction; students in other classes were not required to tell their answer or their confidence to their peer. Finally, the questions appeared at any point during the class period for the cognitive psychology classes, while the questions typically happened at the beginning of each class for the other classes.

## Results

### Analytic strategy

Data are available on the OpenScienceFramework: https://mfr.osf.io/render?url=https://osf.io/5qc46/?action=download%26mode=render.

For most of our analyses we used linear mixed-effects models (Baayen, Davidson, & Bates, 2008; Murayama, Sakaki, Yan, & Smith, 2014). The unit of analysis in a mixed-effect model is the outcome of a single trial (e.g., whether or not a particular question was answered correctly by a particular participant). We modeled these individual trial-level outcomes as a function of multiple fixed effects - those of theoretical interest - and multiple random effects - effects for which the observed levels are sampled out of a larger population (e.g., questions, students, and classes sampled out of a population of potential questions, students, and classes).

Linear mixed-effects models solve four statistical problems involved with the data of peer instruction. First, there is large variability in students’ performance and the difficulty of questions across students and classes. Mixed-effect models simultaneously account for random variation both across participants and across items (Baayen et al., 2008; Murayama et al., 2014). Second, students may miss individual classes and therefore may not provide data across every item. Similarly, classes varied in how many peer instruction questions were posed throughout the semester and the number of students enrolled. Mixed-effects models weight each response equally when drawing conclusions (rather than weighting each student or question equally) and can easily accommodate missing data. Third, we were interested in how several different characteristics influenced students’ performance. Mixed effects models can include multiple predictors simultaneously, which allows us to test the effect of one predictor while controlling for others. Finally, mixed effects models can predict the log odds (or logit) of a correct answer, which is needed when examining binary outcomes (i.e., correct or incorrect; Jaeger, 2008).

We fit all models in R using the lmer() function of the lme4 package (Bates, Maechler, Bolker, & Walker, 2015). For each mixed-effect model, we included random intercepts that capture baseline differences in difficulty of questions, in classes, and in students, in addition to multiple fixed effects of theoretical interest. In mixed-effect models with hundreds of observations, the *t* distribution effectively converges to the normal, so we compared the *t* statistic to the normal distribution for analyses involving continuous outcomes (i.e., confidence; Baayen, 2008). *P* values can be directly obtained from Wald z statistics for models with binary outcomes (i.e., correctness).

### Does accuracy change through discussion?

**Fig. 1:**
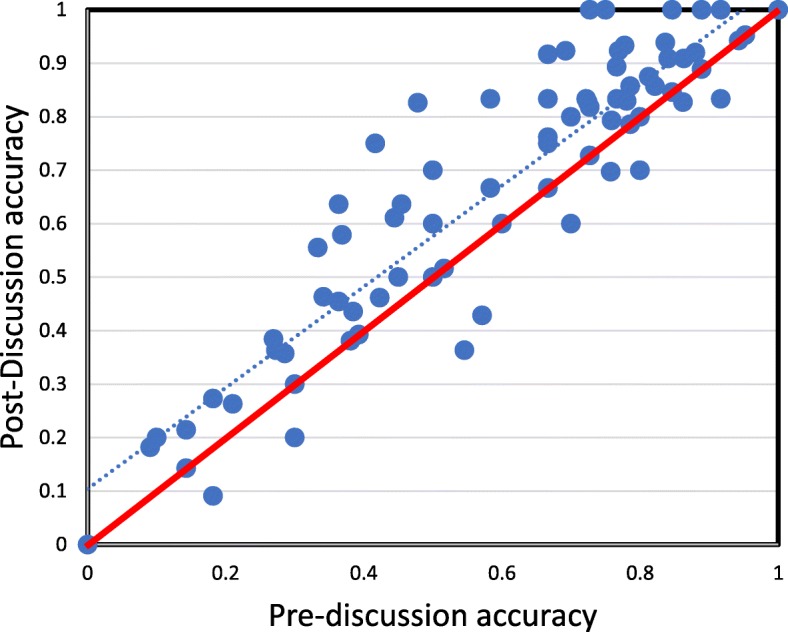
The relationship between pre-discussion accuracy (x axis) and post-discussion accuracy (y axis). Each point represents a single question. The solid diagonal line represents equal pre-discussion and post-discussion accuracy; points above the line indicate improvements in accuracy and points below represent decrements in accuracy. The dashed line indicates the line of best fit for the observed data

First, we examined how correctness changed across peer discussion. A logit model predicting correctness from time point (pre-discussion to post-discussion) revealed that the odds of correctness increased by 1.57 times (95% confidence interval (conf) 1.31–1.87) from pre-discussion to post-discussion, as shown in Table 2. In fact, 88% of students showed an increase or no change in accuracy from pre-discussion to post-discussion. Pre-discussion to post-discussion performance for each class is shown in Table 3. We further examined how accuracy changed from pre-discussion to post-discussion for each question and the results are plotted in Fig. 1. The data show a consistent improvement in accuracy from pre-discussion to post-discussion across all levels of initial difficulty.

**Table 2:** The effect of time point (pre-discussion to post-discussion) on accuracy using a mixed effect logit model

Fixed Effect	$\hat{\beta}$	SE	Wald z	*p*
Intercept	0.68	0.19	3.515	.0004
Time point (pre to post)	0.45	0.09	5.102	< .0001

**Table 3:** Accuracy before and after discussion by class

Class	Pre-correct (mean)	Post-correct (mean)	SD of difference	Paired *t* test	Cohen’s *d*
Cognitive Psych (Psych) 2015	0.67	0.76	0.27	*t* (60) = 2.40, *p* = 0.02	0.31
Cognitive Psych (Psych) 2017	0.65	0.73	0.21	*t* (59) = 2.75, *p* = 0.007	0.36
Decision Making (Ed Psych) 2016	0.57	.66	0.13	*t* (23) = 3.30, *p* = 0.003	0.69
Decision Making (Ed Psych) 2017	0.71	0.75	0.13	*t* (36) = 1.92, *p* = 0.06	0.32
Learning Theories (Ed Psych) 2016	0.58	0.69	0.06	*t* (11) = 5.76, *p* < 0.001	1.74
Learning Theories (Ed Psych) 2018	0.57	0.61	0.09	*t* (13) = 2.00, *p* = 0.07	0.56
Overall	0.65	0.72	0.20	*t* (212) = 5.39, *p* < 0.001	0.37

**Fig. 2:**
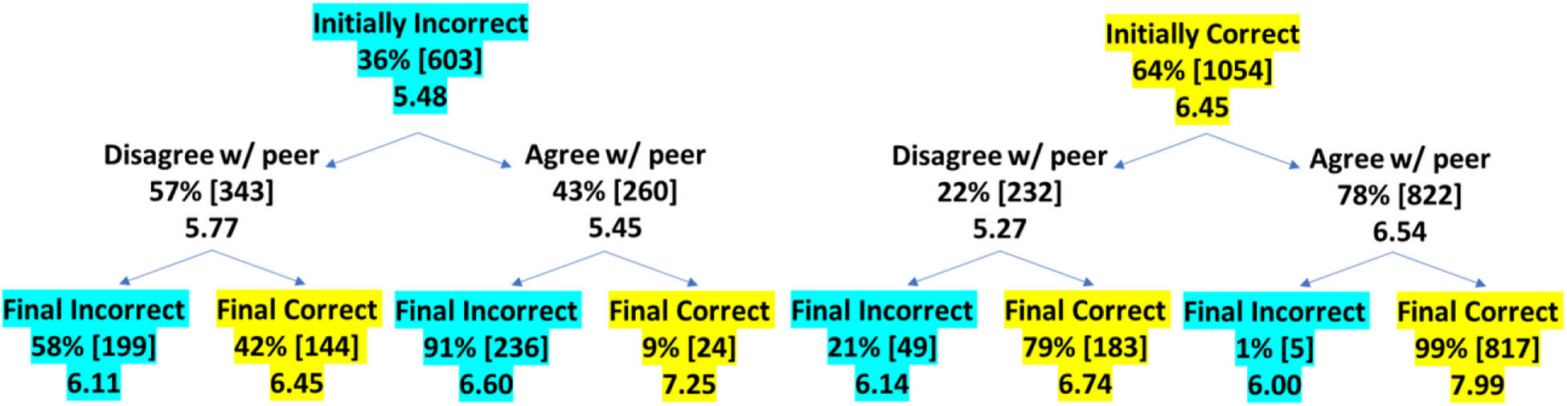
The pathways of answers from pre-discussion (top row) to post-discussion (bottom row). Percentages indicate the portion of items from the category immediately above in that category, the numbers in brackets indicate the raw numbers of items, and the numbers at the bottom of each entry indicate the confidence associated with those items. In the middle, white row, confidence values show the peer’s confidence. Turquoise indicates incorrect answers and yellow indicates correct answers

We examined how performance increased from pre-discussion to post-discussion by tracing the correctness of answers through the discussion. Figure 2 tracks the percent (and number of items) correct from pre-discussion to post-discussion. The top row shows whether students were initially correct or incorrect in their answer; the middle row shows whether students agreed or disagreed with their partner; the last row show whether students were correct or incorrect after discussion. Additionally, Fig. 2 shows the confidence associated with each pathway. The bottow line of each entry shows the students’ average confidence; in the middle white row, the confidence reported is the average of the peer’s confidence.

Broadly, only 5% of correct answers were switched to incorrect, while 28% of incorrect answers were switched to correct following discussion. Even for the items in which students were initially correct but disagreed with their partner, only 21% of answers were changed to incorrect answers after discussion. However, out of the items where students were initially incorrect and disagreed with their partner, 42% were changed to the correct answer.

### Does confidence predict switching?

Differences in the amount of switching to correct or incorrect answers could be driven solely by differences in confidence, as described in our first theory mentioned earlier. For this theory to hold, answers with greater confidence must have a greater likelihood of being correct. To examine whether initial confidence is associated with initial correctness, we calculated the gamma correlation between correctness and confidence in the answer before discussion, as shown in the first column of Table 4. The average gamma correlation between initial confidence and initial correctness (mean (M) = 0.40) was greater than zero, *t* (160) = 8.59, *p* < 0.001, *d* = 0.68, indicating that greater confidence was associated with being correct.

**Table 4:** The gamma correlation between accuracy and confidence before and after discussion for each class

Class	Pre-gamma	Post-gamma	SD of difference	Paired *t* test comparing pre to post^a^	Cohen’s *d*
Cognitive Psych (Psych) 2015	0.60	0.79	0.52	*t* (18) = 1.22, *p* = 0.24	0.29
Cognitive Psych (Psych) 2017	0.27	0.40	0.74	*t* (37) = 2.29, *p* = 0.02	0.38
Decision Making (Ed Psych) 2016	0.36	0.56	0.46	*t* (22) = 3.21, *p* = 0.004	0.47
Decision Making (Ed Psych) 2017	0.47	0.44	0.46	*t* (33) = 0.24, *p* = 0.81	− 0.04
Learning Theories (Ed Psych) 2016	0.18	0.28	0.45	*t* (11) = 1.57, *p* = 0.14	0.23
Learning Theories (Ed Psych) 2018	0.43	0.37	0.37	*t* (13) = 0.58, *p* = 0.57	− 0.16
Overall	0.40	0.48	0.55	*t* (139) = 2.98, *p* = 0.003	0.24

^a^Gamma correlation requires that learners have variance in both confidence and correctness before and after discussion. Degrees of freedom are reduced because many students did not have requisite variation

Changing from an incorrect to a correct answer, then, may be driven entirely by selecting the answer from the peer with the greater confidence during discussion, even though most of the students in our sample were not required to explicitly disclose their confidence to their partner during discussion. We examined how frequently students choose the more confident answer when peers disagree. When peers disagreed, students’ final answers aligned with the more confident peer only 58% of the time. Similarly, we tested what the performance would be if peers always picked the answer of the more confident peer. If peers always chose the more confident answer during discussion, the final accuracy would be 69%, which is significantly lower than actual final accuracy (M = 72%, *t* (207) = 2.59, *p* = 0.01, *d* = 0.18). While initial confidence is related to accuracy, these results show that confidence is not the only predictor of switching answers.

### Does correctness predict switching beyond confidence?

Discussion may reveal information about the correctness of answers by generating new knowledge and testing the coherence of each possible answer. To test whether the correctness of an answer added predictive power beyond the confidence of the peers involved in discussion, we analyzed situations in which students disagreed with their partner. Out of the instances when partners initially disagreed, we predicted the likelihood of keeping one’s answer based upon one’s own confidence, the partner’s confidence, and whether one’s answer was initially correct. The results of a model predicting whether students keep their answers is shown in Table 5. For each increase in a point of one’s own confidence, the odds of keeping one’s answer increases 1.25 times (95% conf 1.13–1.38). For each decrease in a point of the partner’s confidence, the odds of keeping one’s answer increased 1.19 times (1.08–1.32). The beta weight for one’s confidence did not differ from the beta weight of the partner’s confidence, χ^2^ = 0.49, *p* = 0.48. Finally, if one’s own answer was correct, the odds of keeping one’s answer increased 4.48 times (2.92–6.89). In other words, the more confident students were, the more likely they were to keep their answer; the more confident their peer was, the more likely they were to change their answer; and finally, if a student was correct, they were more likely to keep their answer.

**Table 5:** Logit mixed-level regression analysis

Fixed effect	$\hat{\beta}$	SE	Wald z	*p*
Intercept	− 0.18	0.13	1.36	.17
Own confidence (mean-centered)	0.22	0.05	4.16	< .0001
Partner confidence (mean-centered)	−0.18	0.05	3.51	.0005
Own correct	1.50	0.22	6.73	< .0001

The results of a logit mixed level regression predicting keeping one's answer from one's own confidence, the peer's confidence, and the correctness of one's initial answer for situations in which peers initially disagreed

**Fig. 3:**
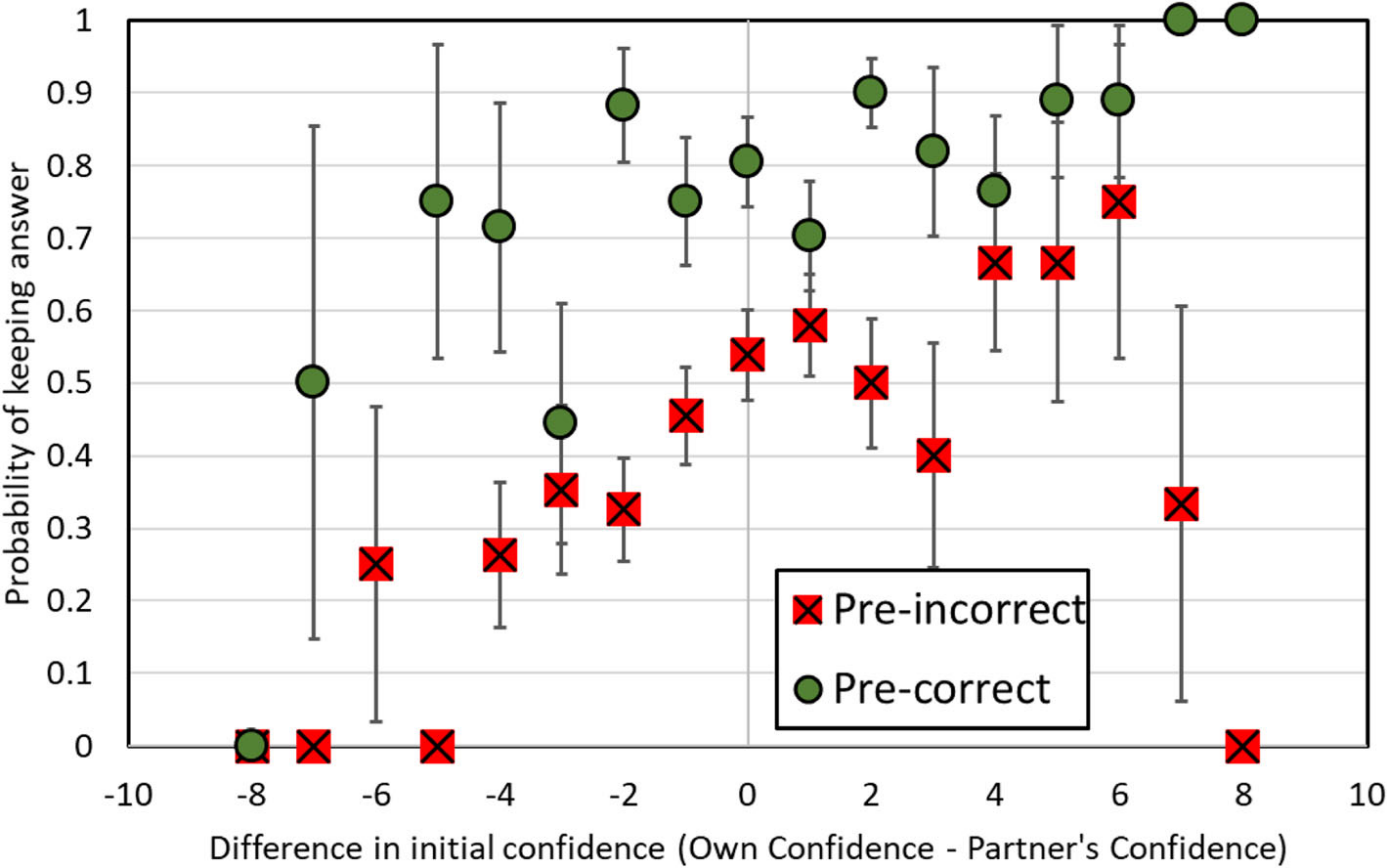
The probability of keeping one’s answer in situations where one’s partner initially disagreed as a function of the difference between partners’ levels of confidence. Error bars indicate the standard error of the proportion and are not shown when the data are based upon a single data point

To illustrate this relationship, we plotted the probability of keeping one’s own answer as a function of the difference between one’s own and their partner’s confidence for initially correct and incorrect answers. As shown in Fig. 3, at every confidence level, being correct led to equal or more frequently keeping one’s answer than being incorrect.

**Fig. 4:**
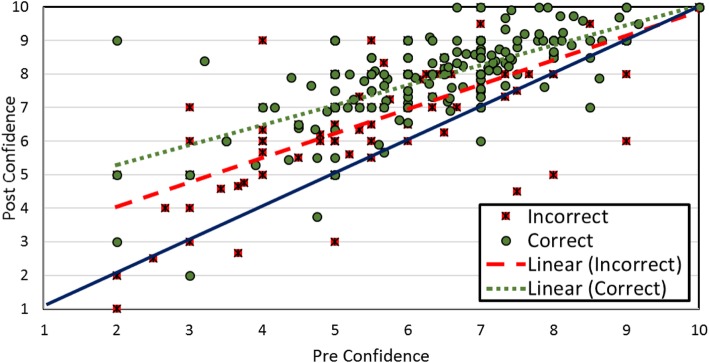
The relationship between pre-discussion and post-discussion confidence as a function of the accuracy of an answer when partners agreed. Each dot represents a student

As another measure of whether discussion allows learners to test the coherence of the correct answer, we analyzed how discussion impacted confidence when partners’ answers agreed. We predicted confidence in answers by the interaction of time point (i.e., pre-discussion versus post-discussion) and being initially correct for situations in which peers initially agreed on their answer. The results, displayed in Table 6, show that confidence increased from pre-discussion to post-discussion by 1.08 points and that confidence was greater for initially correct answers (than incorrect answers) by 0.78 points. As the interaction between time point and initial correctness shows, confidence increased more from pre-discussion to post-discussion when students were initially correct (as compared to initially incorrect). To illustrate this relationship, we plotted pre-confidence against post-confidence for initially correct and initially incorrect answers when peers agreed (Fig. 4). Each plotted point represents a student; the diagonal blue line indicates no change between pre-confidence and post-confidence. The graph reflects that confidence increases more from pre-discussion to post-discussion for correct answers than for incorrect answers, even when we only consider cases where peers agreed.

**Table 6:** Mixed-level regression analysis of predicting confidence

Fixed effect	$\hat{\beta}$	SE	*t* value	*p*
Intercept	5.63	0.21	26.66	
Time point (pre vs post)	1.08	0.14	7.98	< .0001
Initial correct	0.78	0.13	6.05	< .0001
Time Point*Initial correct	0.33	0.15	2.14	.03

The results of the mixed level regression predicting confidence in one's answer from the time point (pre- or post- discussion), the correctness of one's answer, and their interaction for situations in which peers initially agreed

If students engage in more comprehensive answer testing during discussion than before, the relationship between confidence in their answer and the accuracy of their answer should be stronger following discussion than it is before. We examined whether confidence accurately reflected correctness before and after discussion. To do so, we calculated the gamma correlation between confidence and accuracy, as is typically reported in the literature on metacognitive monitoring (e.g., Son & Metcalfe, 2000; Tullis & Fraundorf, 2017). Across all students, the resolution of metacognitive monitoring increases from pre-discussion to post-discussion (*t* (139) = 2.98, *p* = 0.003, *d* = 0.24; for a breakdown of gamma calculations for each class, see Table 4). Confidence was more accurately aligned with accuracy following discussion than preceding it. The resolution between student confidence and correctness increases through discussion, suggesting that discussion offers better coherence testing than answering alone.

## Discussion

To examine why peer instruction benefits student learning, we analyzed student answers and confidence before and after discussion across six psychology classes. Discussing a question with a partner improved accuracy across classes and grade levels with small to medium-sized effects. Questions of all difficulty levels benefited from peer discussion; even questions where less than half of students originally answered correctly saw improvements from discussion. Benefits across the spectrum of question difficulty align with prior research showing improvements when even very few students initially know the correct answer (Smith et al., 2009). More students switched from incorrect answers to correct answers than vice versa, leading to an improvement in accuracy following discussion. Answer switching was driven by a student’s own confidence in their answer and their partner’s confidence. Greater confidence in one’s answer indicated a greater likelihood of keeping the answer; a partner’s greater confidence increased the likelihood of changing to their answer.

Switching answers depended on more than just confidence: even when accounting for students’ confidence levels, the correctness of the answer impacted switching behavior. Across several measures, our data showed that the correctness of an answer carried weight beyond confidence. For example, the correctness of the answer predicted whether students switched their initial answer during peer disagreements, even after taking the confidence of both partners into account. Further, students’ confidence increased more when partners agreed on the correct answer compared to when they agreed on an incorrect answer. Finally, although confidence increased from pre-discussion to post-discussion when students changed their answers from incorrect to the correct ones, confidence decreased when students changed their answer away from the correct one. A plausible interpretation of this difference is that when students switch from a correct answer to an incorrect one, their decrease in confidence reflects the poor coherence of their final incorrect selection.

Whether peer instruction resulted in optimal switching behaviors is debatable. While accuracy improved through discussion, final accuracy was worse than if students had optimally switched their answers during discussion. If students had chosen the correct answer whenever one of the partners initially chose it, the final accuracy would have been significantly higher (M = 0.80 (SD = 0.19)) than in our data (M = 0.72 (SD = 0.24), *t* (207) = 6.49, *p* < 0.001, *d* = 0.45). While this might be interpreted as “process loss” (Steiner, 1972; Weldon & Bellinger, 1997), that would assume that there is sufficient information contained within the dyad to ascertain the correct answer. One individual selecting the correct answer is inadequate for this claim because they may not have a compelling justification for their answer. When we account for differences in initial confidence, students’ final accuracy was better than expected. Students’ final accuracy was better than that predicted from a model in which students always choose the answer of the more confident peer. This over-performance, often called “process gain”, can sometimes emerge when individuals collaborate to create or generate new knowledge (Laughlin, Bonner, & Miner, 2002; Michaelsen, Watson, & Black, 1989; Sniezek & Henry, 1989; Tindale & Sheffey, 2002). Final accuracy reveals that students did not simply choose the answer of the more confident student during discussion; instead, students more thoroughly probed the coherence of answers and mental models during discussion than they could do alone.

Students’ final accuracy emerges from the interaction between the pairs of students, rather than solely from individuals’ sequestered knowledge prior to discussion (e.g. Wegner, Giuliano, & Hertel, 1985). Schwartz (1995) details four specific cognitive products that can emerge through working in dyads. Specifically, dyads force verbalization of ideas through discussion, and this verbalization facilitates generating new knowledge. Students may not create a coherent explanation of their answer until they engage in discussion with a peer. When students create a verbal explanation of their answer to discuss with a peer, they can identify knowledge gaps and construct new knowledge to fill those gaps. Prior research examining the content of peer interactions during argumentation in upper-level biology classes has shown that these kinds of co-construction happen frequently; over three quarters of statements during discussion involve an exchange of claims and reasoning to support those claims (Knight et al., 2013). Second, dyads have more information processing resources than individuals, so they can solve more complex problems. Third, dyads may foster greater motivation than individuals. Finally, dyads may stimulate the creation of new, abstract representations of knowledge, above and beyond what one would expect from the level of abstraction created by individuals. Students need to communicate with their partner; to create common ground and facilitate discourse, dyads negotiate common representations to coordinate different perspectives. The common representations bridge multiple perspectives, so they lose idiosyncratic surface features of individuals’ representation. Working in pairs generates new knowledge and tests of answers that could not be predicted from individuals’ performance alone.

More broadly, teachers often put students in groups so that they can learn from each other by giving and receiving help, recognizing contradictions between their own and others’ perspectives, and constructing new understandings from divergent ideas (Bearison, Magzamen, & Filardo, 1986; Bossert, 1988-1989; Brown & Palincsar, 1989; Webb & Palincsar, 1996). Giving explanations to a peer may encourage explainers to clarify or reorganize information, recognize and rectify gaps in understandings, and build more elaborate interpretations of knowledge than they would have alone (Bargh & Schul, 1980; Benware & Deci, 1984; King, 1992; Yackel, Cobb, & Wood, 1991). Prompting students to explain why and how problems are solved facilitates conceptual learning more than reading the problem solutions twice without self-explanations (Chi, de Leeuw, Chiu, & LaVancher, 1994; Rittle-Johnson, 2006; Wong, Lawson, & Keeves, 2002). Self-explanations can prompt students to retrieve, integrate, and modify their knowledge with new knowledge; self-explanations can also help students identify gaps in their knowledge (Bielaczyc, Pirolli, & Brown, 1995; Chi & Bassock, 1989; Chi, Bassock, Lewis, Reimann, & Glaser, 1989; Renkl, Stark, Gruber, & Mandl, 1998; VanLehn, Jones, & Chi, 1992; Wong et al., 2002), detect and correct errors, and facilitate deeper understanding of conceptual knowledge (Aleven & Koedinger, 2002; Atkinson, Renkl, & Merrill, 2003; Chi & VanLehn, 2010; Graesser, McNamara, & VanLehn, 2005). Peer instruction, while leveraging these benefits of self-explanation, also goes beyond them by involving what might be called “other-explanation” processes - processes recruited not just when explaining a situation to oneself but to others. Mercier and Sperber (2019) argue that much of human reason is the result of generating explanations that will be convincing to other members of one’s community, thereby compelling others to act in the way that one wants.

Conversely, students receiving explanations can fill in gaps in their own understanding, correct misconceptions, and construct new, lasting knowledge. Fellow students may be particularly effective explainers because they can better take the perspective of their peer than the teacher (Priniski & Horne, 2019; Ryskin, Benjamin, Tullis, & Brown-Schmidt, 2015; Tullis, 2018). Peers may be better able than expert teachers to explain concepts in familiar terms and direct peers’ attention to the relevant features of questions that they do not understand (Brown & Palincsar, 1989; Noddings, 1985; Vedder, 1985; Vygotsky, 1981).

Peer instruction may benefit from the generation of explanations, but social influences may compound those benefits. Social interactions may help students monitor and regulate their cognition better than self-explanations alone (e.g., Jarvela et al., 2015; Kirschner, Kreijns, Phielix, & Fransen, 2015; Kreijns, Kirschner, & Vermeulen, 2013; Phielix, Prins, & Kirschner, 2010; Phielix, Prins, Kirschner, Erkens, & Jaspers, 2011). Peers may be able to judge the quality of the explanation better than the explainer. In fact, recent research suggests that peer instruction facilitates learning even more than self-explanations (Versteeg, van Blankenstein, Putter, & Steendijk, 2019).

Not only does peer instruction generate new knowledge, but it may also improve students’ metacognition. Our data show that peer discussion prompted more thorough testing of the coherence of the answers. Specifically, students’ confidences were better aligned with accuracy following discussion than before. Improvements in metacognitive resolution indicate that discussion provides more thorough testing of answers and ideas than does answering questions on one’s own. Discussion facilitates the metacognitive processes of detecting errors and assessing the coherence of an answer.

Agreement among peers has important consequences for final behavior. For example, when peers agreed, students very rarely changed their answer (less than 3% of the time). Further, large increases in confidence occurred when students agreed (as compared to when they disagreed). Alternatively, disagreements likely engaged different discussion processes and prompted students to combine different answers. Whether students weighed their initial answer more than their partner’s initial answer remains debatable. When students disagreed with their partner, they were more likely to stick with their own answer than switch; they kept their own answer 66% of the time. Even when their partner was more confident, students only switched to their partner’s answer 50% of the time. The low rate of switching during disagreements suggests that students weighed their own answer more heavily than their partner’s answer. In fact, across prior research, deciders typically weigh their own thoughts more than the thoughts of an advisor (Harvey, Harries, & Fischer, 2000; Yaniv & Kleinberger, 2000).

Interestingly, peers agreed more frequently than expected by chance. When students were initially correct (64% of the time), 78% of peers agreed. When students were initially incorrect (36% of the time), peers agreed 43% of the time. Pairs of students, then, agree more than expected by a random distribution of answers throughout the classroom. These data suggest that students group themselves into pairs based upon likelihood of sharing the same answer. Further, these data suggest that student understanding is not randomly distributed throughout the physical space of the classroom. Across all classes, students were instructed to work with a neighbor to discuss their answer. Given that neighbors agreed more than predicted by chance, students seem to tend to sit near and pair with peers that share their same levels of understanding. Our results from peer instruction reveal that students physically locate themselves near students of similar abilities. Peer instruction could potentially benefit from randomly pairing students together (i.e. not with a physically close neighbor) to generate the most disagreements and generative activity during discussion.

Learning through peer instruction may involve deep processing as peers actively challenge each other, and this deep processing may effectively support long-term retention. Future research can examine the persistence of gains in accuracy from peer instruction. For example, whether errors that are corrected during peer instruction stay corrected on later retests of the material remains an open question. High and low-confidence errors that are corrected during peer instruction may result in different long-term retention of the correct answer; more specifically, the hypercorrection effect suggests that errors committed with high confidence are more likely to be corrected on subsequent tests than errors with low confidence (e.g., Butler, Fazio, & Marsh, 2011; Butterfield & Metcalfe, 2001; Metcalfe, 2017). Whether hypercorrection holds for corrections from classmates during peer instruction (rather than from an absolute authority) could be examined in the future.

The influence of partner interaction on accuracy may depend upon the domain and kind of question posed to learners. For simple factual or perceptual questions, partner interaction may not consistently benefit learning. More specifically, partner interaction may amplify and bolster wrong answers when factual or perceptual questions lead most students to answer incorrectly (Koriat, 2015). However, for more “intellective tasks,” interactions and arguments between partners can produce gains in knowledge (Trouche et al., 2014). For example, groups typically outperform individuals for reasoning tasks (Laughlin, 2011; Moshman & Geil, 1998), math problems (Laughlin & Ellis, 1986), and logic problems (Doise & Mugny, 1984; Perret-Clermont, 1980). Peer instruction questions that allow for student argumentation and reasoning, therefore, may have the best benefits in student learning.

The underlying benefits of peer instruction extend beyond the improvements in accuracy seen from pre-discussion to post-discussion. Peer instruction prompts students to retrieve information from long-term memory, and these practice tests improve long-term retention of information (Roediger III & Karpicke, 2006; Tullis, Fiechter, & Benjamin, 2018). Further, feedback provided by instructors following peer instruction may guide students to improve their performance and correct misconceptions, which should benefit student learning (Bangert-Drowns, Kulik, & Kulik, 1991; Thurlings, Vermeulen, Bastiaens, & Stijnen, 2013). Learners who engage in peer discussion can use their new knowledge to solve new, but similar problems on their own (Smith et al., 2009). Generating new knowledge and revealing gaps in knowledge through peer instruction, then, effectively supports students’ ability to solve novel problems. Peer instruction can be an effective tool to generate new knowledge through discussion between peers and improve student understanding and metacognition.

## Data Availability

As described below, data and materials are available on the OpenScienceFramework: https://mfr.osf.io/render?url=https://osf.io/5qc46/?action=download%26mode=render.
